# Real-world indoor mobility with simulated prosthetic vision: The benefits and feasibility of contour-based scene simplification at different phosphene resolutions

**DOI:** 10.1167/jov.22.2.1

**Published:** 2022-02-01

**Authors:** Jaap de Ruyter van Steveninck, Tom van Gestel, Paula Koenders, Guus van der Ham, Floris Vereecken, Umut Güçlü, Marcel van Gerven, Yağmur Güçlütürk, Richard van Wezel

**Affiliations:** 1 Department of Artificial Intelligence, Donders Institute for Brain, Cognition and Behaviour, Radboud University, Nijmegen, the Netherlands; 2 Department of Biophysics, Donders Institute for Brain, Cognition and Behaviour, Radboud University, Nijmegen, the Netherlands; 3 Biomedical Signal and Systems, MIRA Institute for Biomedical Technology and Technical Medicine, University of Twente, Enschede, the Netherlands

**Keywords:** prosthetic vision, neuroprosthetics, simulated prosthetic vision, mobility, obstacle avoidance, edge detection, surface boundary detection, deep learning, artificial intelligence

## Abstract

Neuroprosthetic implants are a promising technology for restoring some form of vision in people with visual impairments via electrical neurostimulation in the visual pathway. Although an artificially generated prosthetic percept is relatively limited compared with normal vision, it may provide some elementary perception of the surroundings, re-enabling daily living functionality. For mobility in particular, various studies have investigated the benefits of visual neuroprosthetics in a simulated prosthetic vision paradigm with varying outcomes. The previous literature suggests that scene simplification via image processing, and particularly contour extraction, may potentially improve the mobility performance in a virtual environment. In the current simulation study with sighted participants, we explore both the theoretically attainable benefits of strict scene simplification in an indoor environment by controlling the environmental complexity, as well as the practically achieved improvement with a deep learning-based surface boundary detection implementation compared with traditional edge detection. A simulated electrode resolution of 26 × 26 was found to provide sufficient information for mobility in a simple environment. Our results suggest that, for a lower number of implanted electrodes, the removal of background textures and within-surface gradients may be beneficial in theory. However, the deep learning-based implementation for surface boundary detection did not improve mobility performance in the current study. Furthermore, our findings indicate that, for a greater number of electrodes, the removal of within-surface gradients and background textures may deteriorate, rather than improve, mobility. Therefore, finding a balanced amount of scene simplification requires a careful tradeoff between informativity and interpretability that may depend on the number of implanted electrodes.

## Introduction

Blindness is a common disability that causes impaired daily living functionality and decreases quality of life (Kempen et al., 2012; Stevens, White, & Flaxman, 2013). Among all daily life activities, mobility and obstacle avoidance are often reported to be the most problematic (Van Der Geest & Buimer, 2015). For many cases of blindness, there currently exists no effective treatment. However, neuroprosthetic implants are a promising technology for restoring some form of vision via electrical neurostimulation in the visual pathway (Chen, Wang, Fernandez, & Roelfsema, 2020; Fernández, Alfaro, & González-López, 2020; Lewis et al., 2016; Lewis, Ackland, Lowery, & Rosenfeld, 2015; Riazi-Esfahani et al., 2014; Roelfsema, Denys, & Klink, 2018; Shepherd et al., 2013; Tehovnik & Slocum, 2013; Tehovnik, Slocum, Smirnakis, & Tolias, 2009; Pezaris & Reid, 2007). Using multiple electrodes, such implants can activate a specific arrangement of visual neurons based on camera input. This neural stimulation elicits a perceived pattern of localized point-like flashes of light, referred to as phosphenes, that can be used to represent the surroundings. The greater the number of implanted electrodes, the more phosphenes can be elicited. In this study, we focus on cortical implants that, compared with other types of implants, such as retinal implants, are expected to have a wider range of therapeutic applicability (Fernández, Alfaro, & González-López, 2020), are less amenable to electrical crosstalk (Davis et al., 2012; Wilke et al., 2011), and can accommodate a larger number of electrodes. For instance, recently, Chen, Wang, Fernandez, and Roelfsema (2020) successfully implanted more than one thousand cortical electrodes to achieve artificial visual perception in macaque monkeys.

Although the artificially generated prosthetic percept is relatively limited compared with normal vision, it may provide some elementary perception of the surroundings, re-enabling daily living functionality. For mobility in particular, various studies have investigated the benefits of visual neuroprosthetics in a simulated prosthetic vision (SPV) paradigm with sighted participants. Early work by Cha, Horch, and Normann (1992) used a perforated mask over a CRT monitor to create pixelized vision and demonstrated that 625 simulated phosphenes may provide sufficient information for visually guided mobility. More recent studies report that adequate mobility performance could be achieved with as few as 325 (Srivastava, Troyk, & Dagnelie, 2009) or even just 60 (Dagnelie et al., 2007) phosphenes in a simple environment. Note that a conclusive interpretation of these results is complicated by differences in the mobility task used and the realism of the phosphene simulation.

Besides the number of implanted electrodes, another factor that highly influences the usability of prosthetic implants is the choice of image processing protocol that transfers visual input to an appropriate electrode activation pattern. The translation of complex visual input into a phosphene percept (which by definition is limited) requires an efficient reduction of information and selection of the mere essential visual features for a given task. This can be achieved with the use of traditional computer vision approaches, such as edge detection (Boyle, Maeder, & Boles, 2001; Dowling, Maeder, & Boles, 2004; Guo, Yang, & Gao, 2018), but deep neural network models have also gained increasing interest of prosthetic engineers (e.g., Sanchez-Garcia, Martinez-Cantin, & Guerrero, 2020; Han et al., 2021; Bollen et al., 2019; Bollen, van Wezel, van Gerven, & Güçlütürk, 2019; De Ruyter Van Steveninck, Güçlü, van Wezel, & Van Gerven, 2020; Lozano et al., 2020; Lozano et al., 2018). Various image processing approaches have been proposed for mobility in particular (Barnes et al., 2011; Dagnelie et al., 2007; Dowling, Boles, & Maeder, 2006; Dowling, Maeder, & Boles, 2004; Feng & McCarthy, 2013; McCarthy et al., 2015; McCarthy, Feng, & Barnes, 2013; Parikh, Itti, Humayun, & Weiland, 2013; Srivastava, Troyk, & Dagnelie, 2009; van Rheede, Kennard, & Hicks, 2010; Vergnieux, Mace, & Jouffrais, 2014; Vergnieux, Macé, & Jouffrais, 2017; Zapf, Boon, Lovell, & Suaning, 2016). A main line of research among these studies, is focused on the extraction of geometric structure and object contours for scene simplification. McCarthy et al., for instance, proposed methods for extracting scene structure (McCarthy, Feng, & Barnes, 2013) and surface boundaries (McCarthy, Barnes, & Lieby, 2011) from disparity data. Based on quantitative and qualitative image analysis, the authors suggest that these methods may improve the interpretability of prosthetic vision and could support obstacle avoidance. To behaviorally evaluate the benefits of such scene simplification approaches, Vergnieux et al*.* performed experiments with SPV in a virtual environment (Vergnieux, Macé, & Jouffrais, 2017). The study found that visual simplification decreases virtual wayfinding performance for normal vision, but improves the performance with SPV. The highest performance with SPV was achieved when the scene was reduced to only the surface boundaries (i.e., a wireframe rendering).

The aforementioned literature provides solid evidence that scene simplification, and particularly contour extraction, can help to prevent “overcrowding” (i.e., transmitting more visual features than can be clearly interpreted from the limited phosphene representation) and improves the interpretability of prosthetic vision in a mobility task. Nevertheless, few attempts have been undertaken to empirically test this in a real-world setup, and there are some remaining questions and challenges: first, complex scenes may contain abundant textures and background gradients, which complicate contour extraction with conventional image processing applications. Although previous work has demonstrated that intelligent scene simplification methods may work in basic virtual environments (Vergnieux, Macé, & Jouffrais, 2017) or when evaluated as preconverted images and videos (Sanchez-Garcia, Martinez-Cantin, & Guerrero, 2020; Han et al., 2021), the implementation of a real-time, effective, and practical image processing method in a real-world complex visual environment is a pressing issue that can bring research closer to the clinical situation. Second, it is unclear to what extent scene simplification contributes to improved mobility with SPV. Decreasing the amount of visual information may, on the one hand, increase interpretability by preventing overcrowding, but, on the other hand, excessive deprivation of visual information may also lead to impaired mobility. For example, texture is an important cue that is used in navigation (Gibson, 1950). Explicit investigation of this trade-off between interpretability and informativity for various phosphene resolutions may provide insight into the essential components for visually guided mobility with prosthetic vision.

In the current study, we empirically evaluate contour extraction in a real-world indoor mobility task using a simulation of cortical prosthetic vision. We test two levels of contour-based scene simplification: an edge-based representation, that extracts visual gradients from all areas of the visual scene, versus a stricter surface-boundary representation, in which all within-surface information and background textures are removed. With this comparison in mind, our experiment is designed to address three study aims: i) to explore the restorable benefits for mobility with prosthetic vision and the required number of implanted electrodes; ii) to examine the theoretically attainable benefits of a stricter surface boundary representation by removal of all within-surface gradients and background textures; and iii) to test the feasibility of software-based scene simplification using a pretrained deep neural network architecture for real-time surface boundary detection.

## Materials and methods

### Participants

We recruited 21 participants at the university campus (Radboud University, Nijmegen, the Netherlands) who had no prior experience with simulated phosphene vision. Inclusion criteria were an absence of mobility impairments, low susceptibility to motion sickness, and normal or corrected to normal vision. One participant was unable to perform the experiments owing to virtual reality sickness and was, therefore, excluded from the analysis. Demographics of the remaining 20 participants are displayed in Table 1. The conducted research was approved by the local ethical committee (REC, Radboud University, Faculty of Sciences) and all subjects gave written informed consent to participate.

**Table 1.** Summary of participant characteristics (*n* = 20).

Characteristics	Median	Interquartile range
Age, years	21	20.8–23.3
Height, m	1.84	1.75–1.87

### Experimental setup

**Figure 1.**
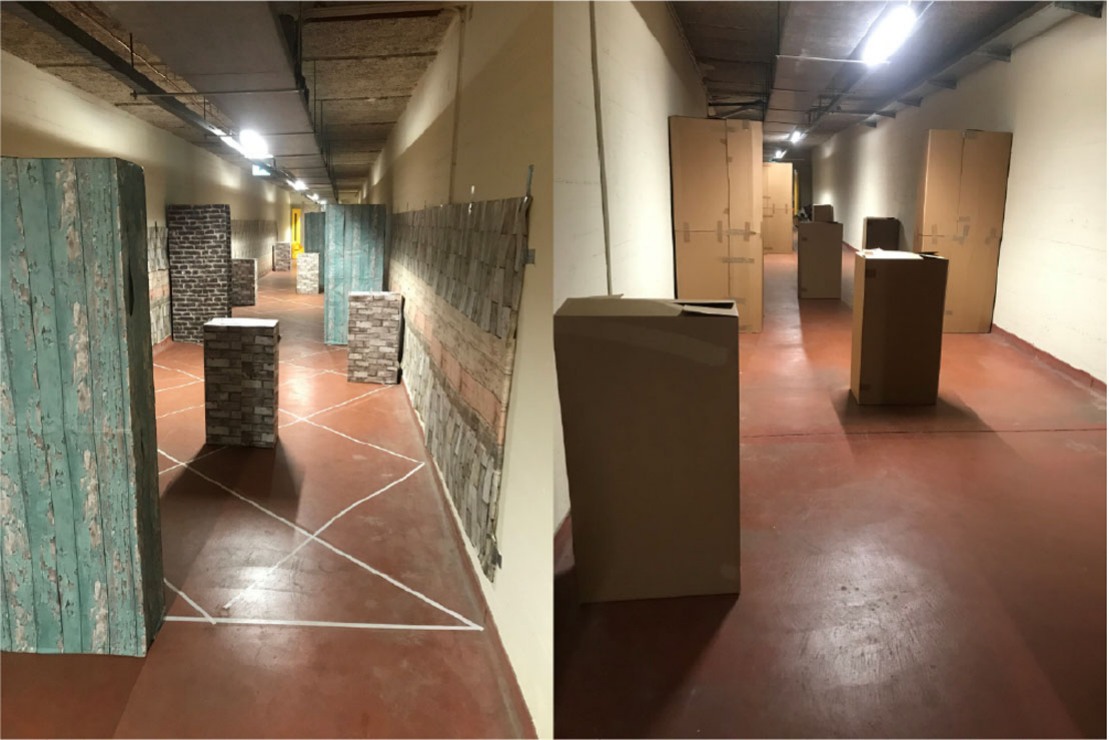
Photos of the complex (left) and plain (right) obstacle course. Both environments contained identical cardboard boxes. In the complex environment, additional visual gradients are created with wallpaper and tape.

**Figure 2.**
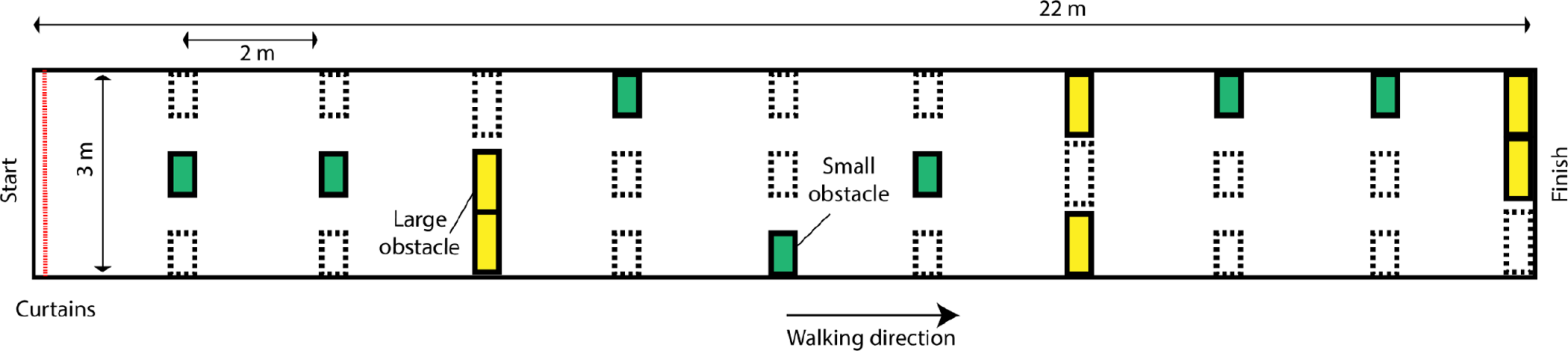
Overview of the obstacle course setup. The yellow boxes indicate large obstacles and the green boxes indicate small obstacles. Dashed lines indicate alternative box locations in other random route permutations. Out of all possible route layouts, a selection of seven routes of similar difficulty (based on the shortest path length around the obstacles) were used, as well as their mirrored versions.

The experiments were situated in a 3-m-wide corridor in the basement of the university building. Two 22-m-long mobility courses were prepared containing seven small (30 × 50 × 90 cm) and six large (30 × 75 × 180 cm) cardboard boxes that were placed along the corridor and acted as obstacles. In one of the two courses, which we refer to as the “complex environment” (as opposed to the “simple environment”); wallpaper and tape were used to provide supplemental visual gradients to the floor, the walls, and the obstacles (Figure 1 and Figure 2). A combination of a laptop (Precision 7550, Dell Technologies) and an attached-by-wire head-mounted virtual reality device (Vive Pro Eye, HTC Corporation) was used for the simulation of prosthetic vision. To eliminate trip hazard, the participant was always accompanied by one of the researchers and connection cables were suspended in the air using a rod. Visual input was captured by the inbuilt frontal camera of the headset and was processed using Python (version 3.6.12) making use of the OpenCV (version 4.4.0) image preprocessing library (Bradski, 2000). During the experiments, a low-quality version of the video input and the displayed phosphene simulation was recorded and saved for post hoc inspection. Trial duration and collisions were registered manually. Furthermore, after each trial, participants were asked to provide a subjective rating on a 10-point Likert scale, indicating to what degree they agreed with the statement that in the current condition it was “easy to walk to the end of the hallway while avoiding the obstacles.” In addition to these primary end points, which were measured for every trial, we also gave participants the opportunity to comment on their general experience in an exit survey. Relevant observations are discussed in the Results.

### Image processing

**Figure 3.**
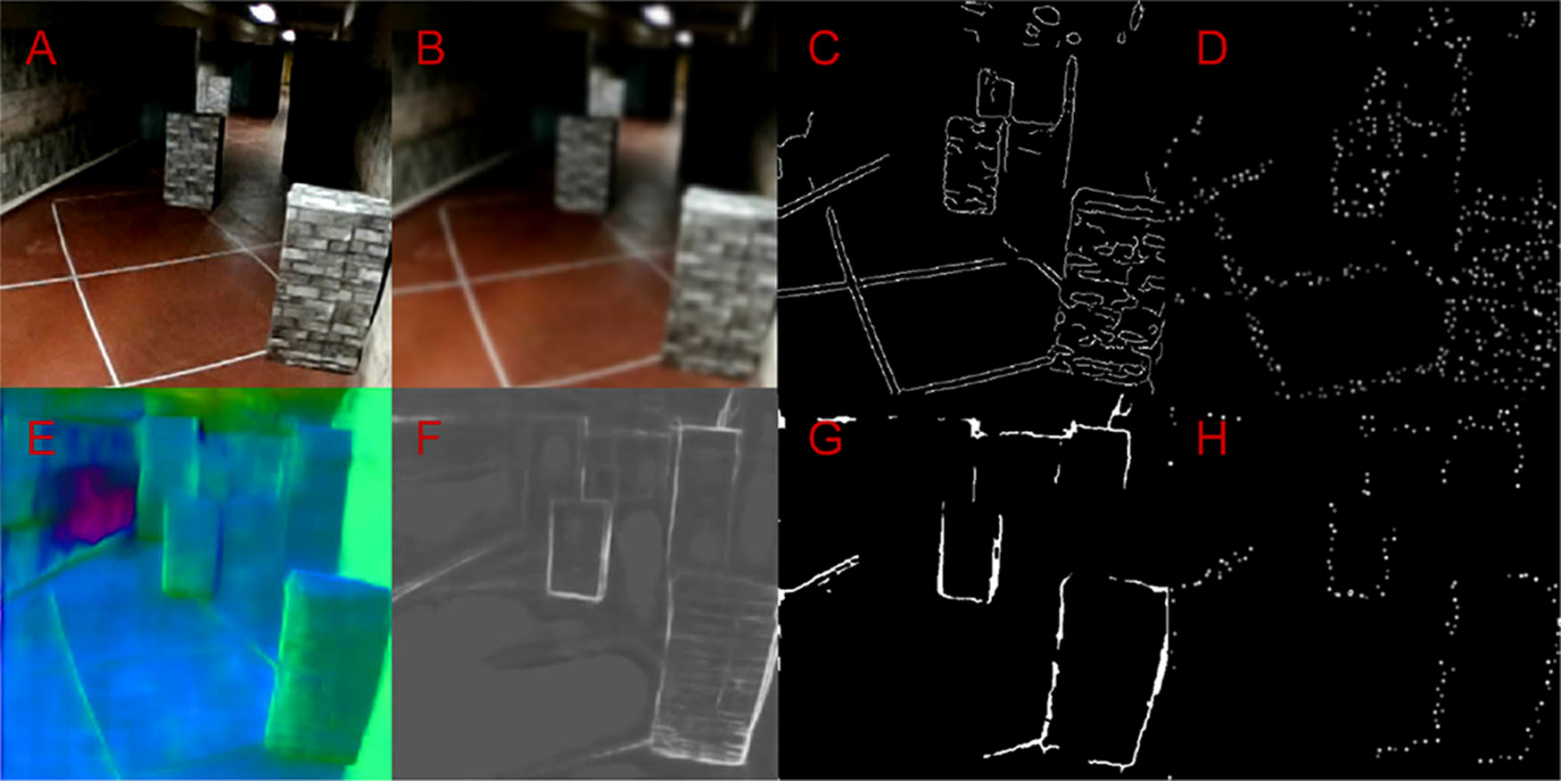
Visualization of the image processing steps. (A) Input image. (B) Blurred image, using Gaussian smooting. (C) Edge mask, produced using the Canny algorithm. (D) Simulated phosphene vision, based on the Canny edge mask. (E) Surface normals prediction by the SharpNet deep learning model. (F) Surface boundary prediction by by the SharpNet deep learning model. (G) Surface boundary mask produced using the SharpNet predictions. (H) Simulated phosphene vision, based on surface boundary mask.

Input frames were obtained from the inbuilt frontal fisheye camera of the virtual reality device. Each frame was processed separately. The frames were cropped and resized to 480 × 480 pixels and depending on the experimental condition, either conventional edge detection was performed with the Canny edge detection (CED) algorithm (Canny, 1986), or surface boundary detection using SharpNet. We used the inbuilt OpenCV CED implementation together with prior smoothing using a two-dimensional Gaussian filter. In the CED algorithm, gradient pixels are accepted as an edge if the gradient is higher than the upper threshold or if the gradient is between the two thresholds and it is connected to a pixel that is above the upper threshold (Canny, 1986). Based on qualitative visual assessment and prior pilot experiments, we determined the optimal lower and higher thresholds for our environment to be equal to 25 and 50 (out of 255), respectively, in combination with a sigma parameter of 3.0 for the Gaussian smoothing. For the surface boundary detection, we used the publicly available implementation of the SharpNet model as described in Ramamonjisoa and Lepetit (2019), which was pretrained on the NYUv2 dataset (Silberman, Hoiem, Kohli, & Fergus, 2012). On our laptop (graphical processing unit: NVIDIA Quadro RTX 4000), this model achieved a framerate of 18.3 Hz (standard deviation, 4.15 Hz) using the PyTorch framework (version 1.6.0) (Mazza & Pagani, 2017). In addition to the raw object boundary prediction, the SharpNet model provides an estimation of depth and surface normals. To achieve optimal results, we combined the contours on the surface normal estimation map with the thresholded boundary prediction, which yielded the best performance in pilot experiments. The optimal threshold for the boundary detection was determined at 94 (out of 255). A visualization of the image preprocessing for one example frame can be found in Figure 3.

### Phosphene simulation

The previous literature reports phosphenes as punctuate dots with a size of 0.2° to 2.0° of visual arc (Bak et al., 1990; Schmidt et al., 1996). In our experiments, phosphenes were simulated as white, equally sized Gaussian blobs of roughly 0.3° of visual arc (sigma, 2.0 pixels) on a rectangular grid in the center of the virtual reality display (480 × 480 pixels; roughly 35° of visual arc). The visual field of our phosphene simulation is kept constant throughout the experiment, as well as the phosphene sizes. The number of phosphenes, however, is varied across study conditions, which means, by consequence, that the phosphene density is also different across study conditions. Note that wherever we refer to the effects of the phosphene resolution, this should be interpreted as the combined effect of the number of phosphenes and the phosphene density. Phosphenes could only take binary values (“on” or “off”), as at this time, cortical visual prostheses do not allow for systematic control over phosphene brightness (Najarpour Foroushani, Pack, & Sawan, 2018; Troyk et al., 2003). To mimic biological irregularities in phosphene mapping, distortion was added to the grid locations and a minor (temporally constant) variation was applied to the brightness of individual phosphenes.

### Experimental procedure

**Figure 4.**
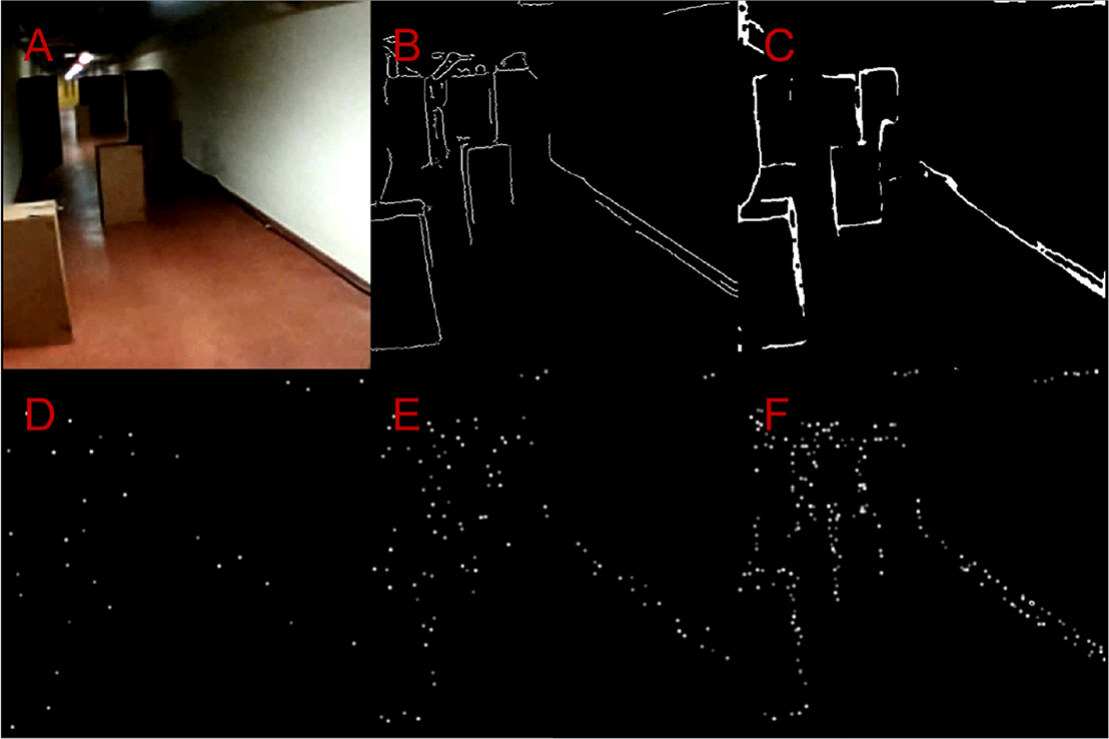
Image processing and phosphene simulation in the plain environment. (A) Input image. (B) Edge mask, produced using the Canny algorithm. (C) Surface boundary mask produced using the SharpNet predictions. (D–F) Comparison of different simulated phosphene resoutions (10 × 10, 26 × 26, and 50 × 50 phosphenes, respectively), with activations based on the Canny edge mask.

The experiment was partitioned into three sessions, starting with a training session (approximately 20 minutes) containing practice trials with the full experimental setup, to allow the participants to get acquainted with the simulated phosphene vision and the experimental task. The remaining two sessions started with two control trials, in which normal vision was simulated by directly displaying the camera input on the virtual reality-device, followed by eight different phosphene conditions. The total duration of the experiment was 2.5 to 3.0 hours. The study conditions were designed to facilitate three types of comparisons, which correspond with our study aims: i) to obtain a general measure of restorability of mobility performance and an indication of the required number of implanted electrodes, we compared the mobility performance with SPV at six different phosphene resolutions with the performance in a control condition with normal camera vision (Figure 4). ii) To examine the theoretically attainable benefits of a stricter surface boundary representation where within-surface gradients and background are removed, we compare the performance with CED in the complex versus the plain visual environment. iii) To assess the feasibility of obtaining such strict scene simplification with real-time deep learning-based surface boundary detection, we tested SharpNet at two different phosphene resolutions in both the complex and plain visual environment. Here, the SharpNet model was evaluated against CED as a control condition. Based on the aforementioned literature on overcrowding, we hypothesize that mobility in the complex environment with a low phosphene resolution (such as 26 × 26 phosphenes), can be improved with deep learning-based scene simplification compared with basic image processing with CED. An overview of the study conditions can be found in Table 2. Note that a representative selection of these conditions was practiced in the training session (i.e., both low and high phosphene resolutions, both environmental complexities, and both image processing methods). At the beginning of each trial, an auditory start cue was presented to the participants. To encourage the maximal performance achievable, as limited by the visual input, instructions were to walk as fast as possible while avoiding the obstacles. Between each trial, the obstacles were systematically shuffled to match one of seven predefined route layouts.

**Table 2.** Overview of study conditions and corresponding number of trials.

Camera vision	CED-based SPV	SharpNet-based SPV
Two trials per session (one for each visual complexity)	Two trials per session for each of the following six phosphene resolutions (one for each visual complexity): • 10 × 10 phosphenes • 18 × 18 phosphenes • 26 × 26 phosphenes • 34 × 34 phosphenes • 42 × 42 phosphenes • 50 × 50 phosphenes	Two trials per session for each of the following two phosphene resolutions (one for each visual complexity): • 26 × 26 phosphenes • 42 × 42 phosphenes
Total (two sessions): 4 trials	Total (two sessions): 24 trials	Total (two sessions): 8 trials

### Randomization

In an effort to minimize systematic bias owing to learning effects, or owing to characteristics of the route layout, both the order of all phosphene simulations and the order of the route layouts were randomized. For corresponding phosphene simulation conditions, the route layouts were matched but mirrored across the two different visual complexity conditions. Similarly, to allow for a clean comparison between the two image preprocessing methods, the route layouts were matched between the SharpNet and corresponding CED conditions.

### Statistical analysis

Statistical analysis was performed using the SciPy statistics toolbox (version 1.3.2) for Python (Virtanen, Gommers, & Oliphant, 2020). All three end point parameters were standardized within participants (i.e., the mean was subtracted and results are divided by the standard deviation) to decrease the variance caused by interindividual differences in walking speed, avoidance strategy, and subjective experience. The end point parameters were found to be non-normally distributed across participants, as assessed with the Shapiro–Wilk test. Statistical hypothesis testing was performed using the Wilcoxon signed-rank test. Alpha was set at 0.05 and adjusted with the Bonferroni method for multiple planned comparisons. Six tests were performed to assess the effect of scene complexity with CED-based SPV at each phosphene resolution. Four tests were performed to compare surface boundary detection with SharpNet against edge detection with CED in each subcondition that was measured (i.e., two phosphene resolutions and two scene complexities).

## Results

### General results

Table 3 provides descriptive statistics for the obtained data. We found a small but significant negative correlation between the trial duration and the trial number (Pearson's R = –0.15; *p* < 0.001). On average, SPV trials in the second session were performed 3.468 seconds faster compared with the first session. No learning effects were found for number of collisions and subjective rating. The average performance varied across participants with a standard deviation of 7.748 seconds for the trial duration, 0.251 for number of collisions, and 0.783 for subjective rating. Regression analysis and subgroup analysis of the average collision frequency did not reveal an effect of trade-off between the number of collisions (accuracy) and trial duration (speed).

**Table 3.** Descriptive statistics of the overall results and the control condition with camera view. Std. = Standard deviation.

	Overall		Control condition	
	Mean	Std.	Mean	Std.
Trial duration (s)	31.02	13.79	16.74	4.438
No. of collisions	0.879	1.526	0	0
Subjective rating	6.130	2.331	9.363	0.660

### Phosphene resolution

**Figure 5.**
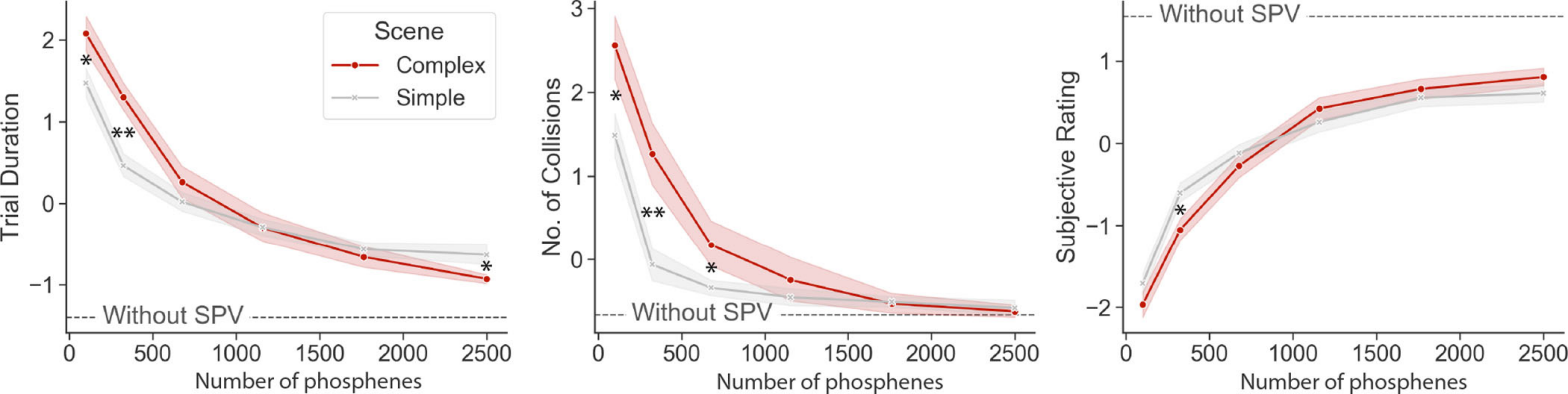
Mobility performance with CED-based SPV. The simulated number of phosphenes is plotted against standardized trial duration (left), standardized number of collisions (middle), and standardized subjective rating (right). Scene complexity is controlled by comparing a simple environment with plain cardboard boxes against a complex environment with additional background and surface textures. The dashed line indicates the average result for the control condition without SPV (i.e., normal camera vision). Asterisk (*) indicates *p* < 0.0125, double asterisk (**) indicates *p* < 0.0025.

The results for the CED trials and the control trials with camera vision are visualized in Figure 5. Assuming an absolute minimal performance at a resolution of 10 × 10 in the complex environment and defining maximal performance as the result obtained with normal vision, more than half the performance is restored at a resolution of 26 × 26 phosphenes (59.2% for trial duration, 90.2% for number of collisions, and 52.6% for subjective rating) in the simple visual environment. At the same resolution of 26 × 26 phosphenes the performance was lower in the complex visual environment (52.2%, 74.1%, and 48.1%, respectively), which is effectively similar to the performance in the simple condition at a lower resolution of 18 × 18 (46.2%, 81.5%, and 38.8%, respectively).

### The effect of scene complexity

The *p* values for the Wilcoxon signed-rank test on the effect of scene complexity with CED are displayed in Table 4. Overall, the complexity-related decrease in performance was found for all lower phosphene resolutions, as evidenced by significant larger trial durations (at resolutions 10 × 10 and 18 × 18), more obstacle collisions (at resolutions 10 × 10, 18 × 18, and 26 × 26) and lower ratings (at resolutions of 10 × 10 and 18 × 18). Notably, in the higher phosphene resolutions this effect was absent or even opposite. With a resolution of 50 × 50 phosphenes, participants achieved a significantly lower trial duration in the complex environment compared with the simple environment.

**Table 4.** *p* Values for Wilcoxon signed rank test for evaluation of the effect of scene complexity with CED. With a Bonferroni correction of α for six planned comparisons, findings are considered significant if *p* < 0.0083.

Resolution	10 × 10	18 × 18	26 × 26	34 × 34	42 × 42	50 × 50
Trial duration	**<0.001**	**<0.001**	0.044	0.765	0.145	**<0.001**
No. of collisions	**0.003**	**<0.001**	**0.004**	0.433	0.889	0.345
Subjective rating	0.039	**0.002**	0.124	0.078	0.221	0.039

### The effect of image processing method: SharpNet versus CED

**Figure 6.**
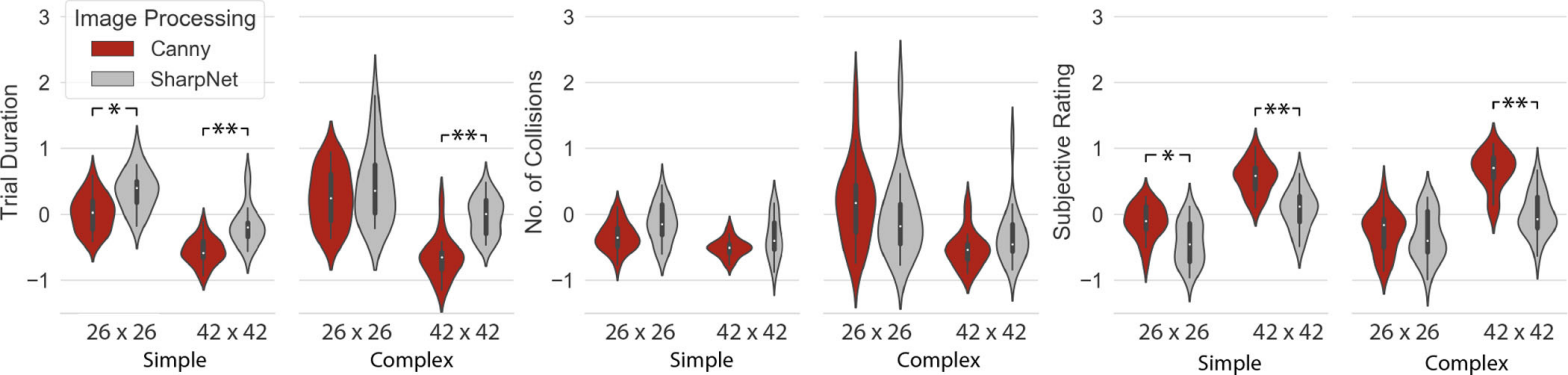
Results of the SharpNet trials with deep-learning based surface boundary prediction versus edge detection with the Canny algorithm. Standardized trial duration (left), standardized number of collisions (middle), and standardized subjective rating (right) are plotted for two phosphene resolutions and two levels of environmental complexity. The complex environment contained additional background and surface textures where the simple environment consisted of plain cardboard boxes. Asterisk (*) indicates *p* < 0.0125, double asterisk (**) indicates *p* < 0.0025.

The *p* values of the Wilcoxon signed-rank test on the effect of image processing are provided in Table 5 and the performance is visualized in Figure 6. For the 42 × 42 phosphenes condition in the complex environment, SharpNet trials were significantly longer (*p* < 0.001) and received a lower subjective rating (*p* < 0.001) compared with CED. In the 26 × 26 phosphenes condition, no significant performance differences were found in the complex environment. In the simple environment performance was worse for the SharpNet condition compared with CED regardless of the phosphenes resolution, as reflected by longer trial durations (*p* < 0.001 for 42 × 42 phosphenes and *p* = 0.005 for 26 × 26 phosphenes) and a lower subjective rating (*p* < 0.001 for 42 × 42 phosphenes, and *p* = 0.004 fors 26 × 26 phosphenes).

**Table 5.** *p* values for Wilcoxon signed-rank test for evaluation of the effect of image processing method. With a Bonferroni correction of α for four planned comparisons, findings are considered significant if *p* < 0.0125.

	Simple scene		Complex scene	
Resolution	26 × 26	42 × 42	26 × 26	42 × 42
Trial duration	**0.005**	**<0.001**	0.478	**<0.001**
No. of collisions	0.055	0.084	0.331	0.169
Subjective rating	**0.004**	**<0.001**	0.520	**<0.001**

### User experience

After the experiment, participants had the opportunity to indicate their personal experience in a survey. Here we report some relevant observations. Of the respondents, 82.4% indicated that they experienced a sufficient amount of practice during the practice session versus 17.6% who indicated that more practice trials would have been beneficial. Upon asking for specific cues that influenced their navigation strategy, 94.1% of the respondents answered that they used the object contours for recognizing obstacles and 47.1% of the respondents indicated that, for some of the trials, choices in navigation were based on chance or intuition rather than understanding of the visual input. Furthermore, 53.0% of the respondents explicitly indicated to make use of apparent differences in lines and textures on the floor, boxes, and walls. In the other comments section, some participants mentioned that the (in)ability to perceive depth strongly influenced the performance for that trial, where one of these participants indicated specifically that forward motion and head movements sometimes contributed to the perception of depth. Some participants indicated that their strategy depended on the number of phosphenes for that trial.

## Discussion

In this study, we evaluated indoor mobility performance with a real-world simulation of prosthetic vision and contour extraction-based image processing. Beside a general evaluation of the restorable mobility performance at different phosphene resolutions, we assessed the inherent, theoretically attainable benefits of decreasing scene complexity via the removal of background textures and within-object gradients. Furthermore, we investigated whether such scene simplification can be achieved practically using a deep neural network approach for surface boundary detection. In this section we provide a discussion on our findings and point out some of the current limitations and directions for future research.

### Mobility with simulated cortical prosthetic vision

The found minimal resolution of 26 × 26 phosphenes for adequate restoration mobility in a simple scene (e.g., 90.2% of obstacle avoidance with normal vision) is comparable with or moderately higher than previous studies that report a minimum of 60 (Dagnelie et al., 2007), 325 (Srivastava, Troyk, & Dagnelie, 2009), or 625 (Cha, Horch, & Normann, 1992) simulated phosphenes. The varying results may be related to the prior experience and amount of practice by the study participants, differences in the mobility task, and the implementation of the phosphene simulation. Dagnelie et al. (2007) found that subjects with previous experience with SPV (≥10 hours) demonstrate improved performance compared with inexperienced subjects, achieving similar results at a lower phosphene resolution. In the experiments by Srivastava, Troyk, and Dagnelie (2009), participants were asked for up to nine laboratory visits. In the current study, despite a majority of participants (82.4%) who indicated to have a sufficient amount of practice, we found a slight but significant improvement in average trial duration over the course of the experiment. This finding means that our results may be influenced by the relatively short exposure to SPV compared with the aforementioned studies. Another factor that may have limited the performance of our participants compared with previous studies is found in the simulation of the phosphenes. In the current study, phosphenes could take binary states (on or off), whereas Dagnelie et al. (2007) and Srivastava, Troyk, and Dagnelie (2009) used eight or four levels of grayscale intensities, respectively. Although some relationship between stimulation parameters and phosphene size has been established (Brindley & Lewin, 1968), at this time cortical visual prostheses do not allow for systematic control over phosphene brightness (Najarpour Foroushani, Pack, & Sawan, 2018; Troyk et al., 2003). The current simulation with binary phosphenes, therefore, provides a valuable addition to previous literature that do not implement this constraint. Note that the field is developing rapidly and results from further clinical work can guide SPV research for the development of realistic phosphene simulations, which, vice versa, can accelerate clinical developments by answering fundamental questions about prosthetic design (Najarpour Foroushani, Pack, & Sawan, 2018). The curves in Figure 5 suggest that—maybe unsurprisingly—even at higher phosphene resolutions there remains a gap between SPV and normal vision. This implies that, besides increasing the number of electrodes, there are other challenges to be taken before prosthetic vision approaches the quality of normal sight. Even with the current technological prospects, there are many design choices that influence the usefulness. For example, in experiments with SPV, Cha, Horch, and Normann (1992) demonstrated that, in line with other low-vision research (Marron & Bailey, 1982), the distribution of simulated phosphenes across the visual field can have an impact on mobility. Future studies with SPV could further explore the impact of using different electrode locations in the visual cortex on the mobility performance.

### The effect of visual complexity

Our results demonstrate that scene simplification via the removal of background textures and within-object gradients may improve mobility performance at lower phosphene resolutions. In the higher phosphene resolutions, this effect was absent or even opposite, indicating an interaction between phosphene resolution and visual complexity. On the one hand, these findings confirm previous suggestions that low-resolution prosthetic vision quickly gets overcrowded (Vergnieux, Macé, & Jouffrais, 2017). A post hoc inspection of the simulated prosthetic percept, as well as the responses on the exit interview, revealed that overabundant phosphene activity renders it almost impossible to distinguish the floor, walls, and objects. In other words, at low phosphene resolutions visual complexity comes at the cost of interpretability. At the same time, excessive removal of visual information at higher phosphene resolutions may negatively influence the visual processing abilities that are required for mobility. Optic flow processing, for instance, which depends on dynamic tracking of local visual patterns, is an important requirement for the estimation of ego-motion and heading (Lappe, Bremmer, & van den Berg, 1999; Warren et al., 2001). Especially in low-vision conditions, and even phosphene vision, the removal of optical flow cues may have a negative impact on the recognition of scene structure and foreground objects (Pan & Bingham, 2013; Qiu et al., 2018). Besides serving as dynamic cues for optic flow perception, surface information, and background textures may have also directly contributed to the detection of foreground objects, by facilitating figure-ground segregation (Caputo, 1996; Machilsen & Wagemans, 2011). Note that these specific interpretations regarding the underlying visual processing remain somewhat speculative, because the downstream visual processing of phosphene vision has been studied sparsely. Yet, based on the performance measures and the responses from the exit survey, we can conclude that the representation of background textures and surface gradients at higher resolutions of prosthetic vision proves to be informative for mobility—at least in the current environment. Combined, these conclusions advocate for a balanced compromise between informativity and interpretability. The optimal amount of scene simplification should be a careful choice that depends on the characteristics of the implant and environmental context.

### Feasibility of deep learning-based surface boundary detection for scene simplification

Besides the theoretically attainable benefit of strict scene simplification, which was tested by removal of gradients and background textures in the environment, we evaluated its practical feasibility through intelligent image processing. Comparing SharpNet with CED, no significant improvement in performance was found in any of the study conditions. Looking at the results in the complex environment, the relative decreased performance with SharpNet in the higher phosphene resolution (42 × 42 phosphenes) is in line with the aforementioned analysis of the CED trials and suggests that that removal of gradients and background textures may not be beneficial at higher resolutions. The absence of improvement in the SharpNet trials with a lower phosphene resolution (26 × 26 phosphenes), however, is unexpected; as for low resolutions, a strict scene simplification method is theorized to prevent overcrowding of the phosphene representation (Vergnieux, Macé, & Jouffrais, 2017). Even more unpredicted is the omnipresent performance decrease in the plain environment, because in this environment the behavior of the SharpNet model is expected to be similar to CED—all visual gradients, besides shadows, match object surface boundaries. Rather than the inherent disadvantages of surface boundary detection, these findings are likely to be explained by poor achievement of the current implementation. Here we summarize a few potential issues. First, a post hoc inspection of the captured image stream revealed poor prediction of the surface boundaries by the SharpNet model. Please note that the output of CED on images acquired in the plain environment (Figure 4B) is effectively equivalent to the ideal output of SharpNet in the complex environment (compare with Figure 3G). The network is trained on a naturalistic indoor image dataset and the underperformance may reflect poor generalizability to the current environment. Furthermore, based on incidental reports from the exit survey and a post hoc visual inspection of the videos, head movements seemed to negatively influence the prediction performance, indicating that the network might be sensitive to motion blur. This factor occasionally caused obstacles to remain undetected. Second, a potential problem with the current SharpNet implementation is that it is based on individual frame processing, resulting in large frame-to-frame differences. The lack of dynamic consistency may have cause a decreased interpretability of the phosphene representations. Third, participants might have experienced difficulties adjusting between the two image processing strategies. Although participants were trained during the practice session, in equal amounts for both methods, by design, our experiment contained fewer SharpNet trials compared with CED trials. This relative underrepresentation may have caused decreased familiarity with the SharpNet condition. Last, the current image processing pipeline is to some extent based on arbitrary choices. Although the effects of specific parameter settings and processing choices were evaluated visually and tested in behavioral pilot experiments, it might be the case that a different configuration would yield better results. The absence of improvement with the deep learning based image processing for prosthetic vision is regrettable, given the potential that was demonstrated in other tasks such as object recognition (Han et al., 2021; Sanchez-Garcia, Martinez-Cantin, & Guerrero, 2020) and emotion recognition (Bollen et al., 2019; Bollen, van Wezel, van Gerven, & Güçlütürk, 2019). Other types of preprocessing approaches have been proposed for mobility in particular, including depth-based (Barnes et al., 2011; McCarthy et al., 2015) or contour-based (Dowling, Maeder, & Boles, 2004; McCarthy, Feng, & Barnes, 2013; Vergnieux, Macé, & Jouffrais, 2017) rendering, where a strict surface boundary-based (i.e., “wireframe”) representation was found to be most effective in virtual mobility experiments (Vergnieux, Macé, & Jouffrais, 2017). Although the results from our physical scene simplification comparison (see previous section) support the value of this approach for lower phosphene resolutions, a feasible real-time implementation for prosthetic vision remains to be realized.

### Limitations and future directions

To our knowledge, our study is the first that directly parametrized visual complexity in a real-world mobility experiment with SPV, by adding textures on objects floors and walls. Despite these efforts in addressing real-world visual complexity of indoor environments, the current scene is still a controlled version of visual navigation. A next step toward the assessment of the requirements of SPV for daily life mobility would require free navigation in interactive environments, with realistic objects and visual cues for orientation. Future work could also further investigate other more specific mobility-related problems such as stair climbing, curb following, or avoidance of elevated objects. All of these situations may be relevant to the clinical target population. Furthermore, note that the navigation strategies that were used by the sighted participants in this simulation study may not capture some aspects of clinical reality. For instance, long-term cane users or people who have undergone blind rehabilitation may be very proficient in the use of nonvisual cues, such as haptic or auditory signals. It is also important to consider potential differences in perceptual learning between sighted and blind individuals, owing to cortical reorganization (Horton, Fahle, Mulder, & Trauzettel-Klosinski, 2017). Furthermore, we did not measure eye movements and the participants in our study were unrestricted in making saccades for exploring the rendered scene. With the current state of the technology, this will not be the case for prosthetic vision users. The extent to which our results and those from other SPV studies extrapolate to visual prosthetic users remains to be tested in clinical studies. Another limiting aspect of our study concerns the realism of the phosphene simulation. Our SPV model of cortical prosthetic vision simulates a hypothetical, future visual prosthesis capable of producing idealized (small, circular, nonoverlapping) phosphenes. Our simulation did not incorporate the effects of cortical magnification Brindley and Lewin, 1968; (Srivastava, Troyk, & Dagnelie, 2009), where size and spacing are known to increase as a function of foveal eccentricity. The same holds for dynamic effects such as phosphene fading and interactions when stimulating neighboring electrodes (Brindley & Lewin, 1968; Dobelle, Mladejovsky, & Girvin, 1974). Similar to this retinal equivalent by Beyeler, Boynton, Fine, and Rokem (2017), an interesting line of future work could focus on the development of more realistic perceptual models of cortical prosthetic vision. Last, some previous studies investigated the effects of scene simplification compared with direct grayscale pixel intensity mapping (e.g., Sanchez-Garcia, Martinez-Cantin, & Guerrero, 2020; Vergnieux, Macé, & Jouffrais, 2017), a limitation of our study is that we did not include this condition in our experiments. Including grayscale intensity mapping as a study condition would have enabled us to compare the effect of contour-based scene simplification to a less-restricted control condition with SPV.

## Conclusion

Investigating suitable computer vision strategies for scene simplification is an important step in the development of visual prostheses. Our results suggest that contour-based SPV with a resolution of 26 × 26 phosphenes provides adequate information for mobility. Strict scene simplification with surface boundary extraction may help to overcome visual overcrowding at lower phosphene resolutions. However, the presence of within-surface information and background textures improves performance at higher phosphene resolutions. Therefore, choosing a balanced amount of information reduction is advised, depending on the number of implanted electrodes. Currently, the implementation of deep learning models for surface-boundary detection in a real-time mobility task remains challenging and future research and empirical validation is required to further explore the potential of this approach.
